# Chronic Maternal Vitamin B12 Restriction Induced Changes in Body Composition & Glucose Metabolism in the Wistar Rat Offspring Are Partly Correctable by Rehabilitation

**DOI:** 10.1371/journal.pone.0112991

**Published:** 2014-11-14

**Authors:** Kalle Anand Kumar, Anumula Lalitha, Umakar Reddy, Giriraj Ratan Chandak, Shantanu Sengupta, Manchala Raghunath

**Affiliations:** 1 Division of Endocrinology and Metabolism, National Institute of Nutrition, Indian Council of Medical Research (ICMR), Hyderabad, Telangana, India; 2 Centre for Cellular and Molecular Biology (CCMB), Council of Scientific and Industrial Research (CSIR), Hyderabad, Telangana, India; 3 Institute of Genomics and Integrative Biology, Council of Scientific and Industrial Research (CSIR), New Delhi, India; University of Missouri, United States of America

## Abstract

Maternal under-nutrition increases the risk of developing metabolic diseases. We studied the effects of chronic maternal dietary vitamin B12 restriction on lean body mass (LBM), fat free mass (FFM), muscle function, glucose tolerance and metabolism in Wistar rat offspring. Prevention/reversibility of changes by rehabilitating restricted mothers from conception or parturition and their offspring from weaning was assessed. Female weaning Wistar rats (n = 30) were fed *ad libitum* for 12 weeks, a control diet (n = 6) or the same with 40% restriction of vitamin B12 (B12R) (n = 24); after confirming deficiency, were mated with control males. Six each of pregnant B12R dams were rehabilitated from conception and parturition and their offspring weaned to control diet. While offspring of six B12R dams were weaned to control diet, those of the remaining six B12R dams continued on B12R diet. Biochemical parameters and body composition were determined in dams before mating and in male offspring at 3, 6, 9 and 12 months of their age. Dietary vitamin B12 restriction increased body weight but decreased LBM% and FFM% but not the percent of tissue associated fat (TAF%) in dams. Maternal B12R decreased LBM% and FFM% in the male offspring, but their TAF%, basal and insulin stimulated glucose uptake by diaphragm were unaltered. At 12 months age, B12R offspring had higher (than controls) fasting plasma glucose, insulin, HOMA-IR and impaired glucose tolerance. Their hepatic gluconeogenic enzyme activities were increased. B12R offspring had increased oxidative stress and decreased antioxidant status. Changes in body composition, glucose metabolism and stress were reversed by rehabilitating B12R dams from conception, whereas rehabilitation from parturition and weaning corrected them partially, highlighting the importance of vitamin B12 during pregnancy and lactation on growth, muscle development, glucose tolerance and metabolism in the offspring.

## Introduction

Under-nutrition continues to be a major problem in the developing world, particularly among women and children. Its adverse health effects are frequently compounded by deficiencies of micronutrients: iodine, iron, zinc, vitamin A, folic acid and vitamin B12 in particular. Chiun et al [1] reported that vitamin B12 administration to young vitamin B12 deficient female rats increased food intake, enhanced their growth rate and suggested that it may play a role in carbohydrate or fat metabolism. Conditions in the maternal womb program fetal physiology [2] and nutrition is the major intrauterine environmental factor that alters the expression of the fetal genome which may have life-long consequences. Indeed, Emerson et al [3] showed that maternal vitamin B12 deficiency decreased litter size and birth weight of the offspring compared to control diet fed rats.

Vitamin B12 is essential for neural development, myelination of nervous system and its deficiency results in neurological complications [4]. It is an important component of one carbon metabolism which modulates methylation of DNA and proteins [5]. Its deficiency increases plasma homocysteine, an independent risk factor for recurrent, spontaneous, early pregnancy losses [6]. That in patients with metabolic syndrome, folate and vitamin B12 treatment improved insulin resistance (IR) and endothelial dysfunction, in addition to decreasing homocysteine levels, suggests its importance to metabolic syndrome [7]. However, the role of maternal vitamin B12 deficiency in the development of adiposity, IR and associated diseases like type 2 diabetes (T2D) and cardio vascular diseases (CVD) in the offspring is not known.

We recently reported low birth weight in the offspring born to vitamin B12 restricted (but not folate or folate and vitamin B12 dual restricted) rat dams and that offspring of folate and/or vitamin B12 restricted rat dams weighed higher than controls at/from weaning [8]. They had higher body fat% (especially visceral fat) at/from three months of age and at 12 months had altered lipid metabolism, adipocytokine levels and were dyslipidemic. Lawlor [9] reported that birth weight of the offspring is inversely related to maternal insulin resistance and we observed earlier that after 3 months of dietary vitamin B12 restriction, female Wistar rats had increased body fat% and dyslipidemia [8], which usually precede IR. It therefore appears that B12R dams may have developed IR during pregnancy and hence the lower birth weight of the offspring.

Considering that during fetal growth, decreased muscle and soft tissue development is compensated by/associated with increased body fat [10] and we reported similar findings earlier in the offspring of rat dams deficient in a variety of micronutrients [11]–[15], it was considered pertinent to assess changes in the LBM and FFM in the B12R offspring and their reversibility/prevention by rehabilitation.

Growing evidence suggests that epigenetics links genetics and environment in framing endocrine function [16] and that maternal nutritional status can alter the epigenetic state of the fetal genome and imprint gene expression [17]. Epigenetic alterations in early embryos may be carried forward to subsequent developmental stages [18]. DNA methylation, histone tail modification and chromatin remodeling are the known mechanisms mediating epigenetics which affect gene expression [19]. Kevin et al [20] reported that insulin resistance and blood pressure in offspring are determined by maternal folate and methionine status during the peri-conceptional periods that would lead to epigenetic alterations like DNA methylation. Indeed, Michal et al [21] reported that pre or peri-conceptional supplementation of folic acid and vitamin B6 to mothers with history of fetal growth restriction and hyperhomocytenemia resulted in favorable perinatal outcome.

Considering that i) vitamin B12 deficiency is a significant public health problem among Indians [22], ii) maternal vitamin B12 restriction impaired body adiposity and adipose tissue function in rat offspring [8] and iii) adipose and muscle are the two insulin sensitive tissues primarily responsible for the postprandial clearance of glucose [23] we have now assessed the effects of chronic maternal vitamin B12 restriction on the development and function of muscle in Wistar rat offspring. In addition, prevention/reversibility of the changes by appropriate rehabilitation and the role if any of altered oxidative stress and antioxidant status in the changes seen in offspring were also evaluated.

## Materials and Methods

### Ethics statement

The experiment was conducted in outbred Wistar rats at the animal house in Centre for Cellular and Molecular Biology, Hyderabad, India, in accordance with ‘principles of laboratory animal care’ [24] and with the approval of the ‘Institute’s Ethical Committee on Animal Experiments’.

### Justification for using a Wistar rat model

Rodents have been important models in fetal and neonatal studies because of the strong similarities between murine and human placentae [25]. Conventionally, albino rats have been in use to assess the effects of nutrition on development, physiology and function [25]. Therefore, we have used the Wistar rat model to assess the effects of maternal vitamin B12 restriction on the development and function of muscle (LBM) and impairment if any in IR, glucose tolerance and metabolism.

### Feeding and maintenance of animals

**Figure 1:**
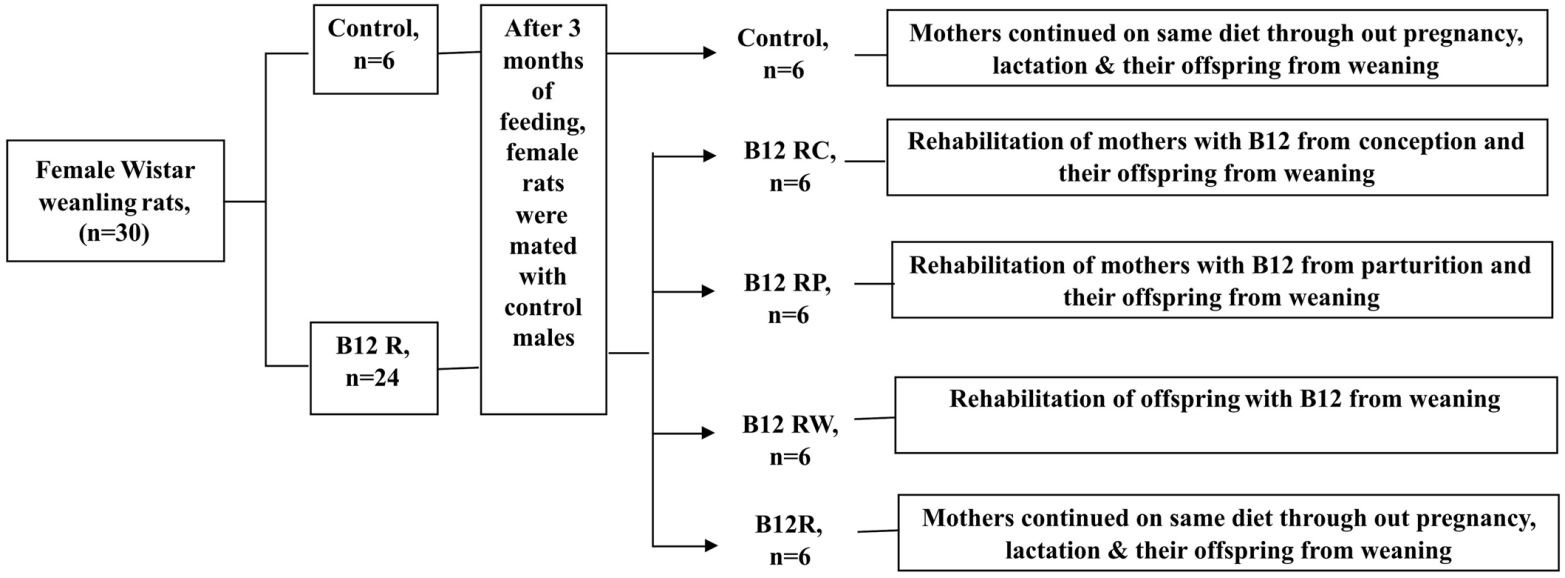
Feeding protocol for different groups of Wistar female rats and their male offspring. Schematic representation of the feeding protocol. Control (C), B12 restricted (B12R), B12 rehabilitation from conception (B12RC), B12 rehabilitation from parturition (B12RP), B12 rehabilitation from weaning (B12RW).

The study design of the animal experiment is given schematically in figure 1. Outbred, weanling, female Wistar rats (n = 30) were housed individually in polypropylene cages with wire mesh bottom and maintained at 22°C±2, under standard lighting conditions (12- hour light/dark cycle). The animals were divided in to two groups and fed *ad libitum* for 12 weeks, a casein based (20% protein) (AIN 76 A) control diet (n = 6) or the same diet restricted in vitamin B12 (B12R) (n = 24). The control and vitamin B12 restricted diets were purchased from M/S Research Diets Inc., USA and all animals had free access to deionized water. Vitamin B12 content of the restricted diet was approximately 60% of that of control diet (0.006 vs 0.010 mg/kg diet); B12R diet contained 50 g pectin/kg diet (in addition to cellulose), because it has been shown earlier that pectin binds vitamin B12 in the intestine and makes it less bioavailable [26]. Daily food intake and weekly body weights were monitored till 12 weeks of feeding and their plasma folate, vitamin B12 and homocysteine levels were determined at the end of 12 weeks of feeding.

### Mating of animals, pregnancy, lactation and weaning

After ensuring deficiency in B12R rats (low plasma vitamin B12 levels), they were mated with control males (@ two females per male). The day on which a vaginal plug was detected was counted as day 1 of conception/pregnancy and six pregnant B12R rats were rehabilitated with control diet from conception and their offspring were weaned on to control diet (B12RC). The remaining B12R dams (n = 18) continued on restricted diet throughout pregnancy and at/from parturition, six B12R dams were switched over to control diet and their offspring were weaned on to control diet (B12RP). At/from weaning, offspring born to six B12R dams were switched to control diet (B12RW), while the offspring born to the remaining six B12R dams continued on restricted diet (B12R). To ensure comparable nutrition to the offspring in all the groups, a uniform litter size of eight pups (equal number of males and females wherever possible) was maintained with each mother from day 3 of lactation till weaning on postnatal day 21. Considering that estrogens influence insulin action, glucose homeostasis and insulin sensitivity [27]–[29], we have monitored the effects in only the male offspring (n = 24 for each group) to avoid the interference of estrogens on the parameters studied and to assess the effects of maternal vitamin B12 restriction *per se* in the offspring. The plasma homocysteine and vitamin B12 status were monitored in the offspring at quarterly intervals starting from 3 months of their age.

### Body composition

Body composition was determined as described by us earlier [11], [12], in the female Wistar rats after three months of feeding their respective diets (just before mating) and in the offspring at 3,6,9 and 12 months of their age using total body electrical conductivity (TOBEC), a small animal body composition analysis system (model SA 3000 multi-detector; EMSCAN, Springfield, IL). Lean body mass% (LBM%) and fat free mass% (FFM%) were computed according to Morbach and Brans [30]. Difference between the LBM% and FFM% was calculated and considered to reflect the percent of tissue associated fat (TAF%).

### Biochemical parameters

Plasma vitamin B12 levels were determined by RIA kit (SIEMENS Medical Solutions Diagnostics) based on a dual count, solid phase no boil assay and homocysteine concentrations were analyzed in plasma by HPLC equipped with a fluorescence detector as described earlier [31].

#### i) Glucose metabolism

- Fasting glucose, insulin and insulin resistance: To assess the animals’ IR status, they were fasted overnight and blood samples were collected from tail vein. Plasma glucose was estimated using an enzymatic kit (glucose-oxidase/peroxidase kit, Biosystems, Spain); plasma insulin using the RIA kit (for rat insulin from Linco Research, USA) and HOMA IR was computed as mentioned earlier [11], [12] in the offspring at 3,6,9 and 12 months of their age.
- Oral glucose tolerance and post prandial insulin resistance: At the time points mentioned above, oral glucose tolerance test was conducted in six rats (fasted overnight) from each group, by administering through oral gavage, a glucose solution (40 g/dl) @ 2.5 g/Kg body weight and blood samples were drawn before and at 30, 60 and 120 minutes after the glucose load. Plasma glucose and insulin were determined and the rats’ insulin response to glucose challenge and its post-prandial insulin resistance were evaluated from the area under curve (AUC) for insulin and the ratio of AUC glucose to AUC insulin as described by us earlier [11], [12].
- Basal and insulin stimulated glucose uptake by muscle (diaphragm): Diaphragm is considered a skeletal muscle rather than smooth muscle because the cells cannot transfer nerve impulses from one to another and each cell is indeed connected to a neuron [32]. Also, GLUT1 (in membrane) and GLUT4 (in intracellular compartments), the two glucose transporters important in postprandial blood glucose clearance, are expressed both in skeletal muscle and diaphragm and diaphragm has been used commonly for determining glucose uptake by skeletal muscle [33], [34]. Therefore in the present study, diaphragm has been used to study the effect of maternal vitamin B12 restriction on glucose uptake (basal and inulin stimulated) by skeletal muscle in the offspring. Briefly, intact diaphragm was isolated from the offspring of different groups at the time points mentioned earlier and the uptake of ^3^H labeled 3-O- methyl D glucose (basal and insulin stimulated) was determined according to Kipnis and Cori [35] as described by us earlier [36].
- Intra cellular glucose metabolism: To assess whether increased gluconeogenesis underlies the high fasting glucose levels in B12R offspring, we determined the activities of the key, rate limiting enzymes: glucokinase (GK), pyruvate-kinase (PK), fructose-1,6-bisphosphatase (FBPase) and phosphoenolpyruvate-carboxykinase (PEPCK) in the cytosolic fraction of liver using standard methods [37]–[40]. Activity of glucose 6 phosphatase (G6Pase) was determined in the microsomal fraction (100,000 g pellet) according to Gierow and Jergil [41].

#### ii) Determination of oxidative stress

Considering that i) Urakawa [42] proposed greater oxidative stress and/or decreased anti-oxidant status to be one of the mechanisms by which obesity, insulin resistance and metabolic syndrome lead to T2DM in humans and ii) we observed them earlier to be associated with maternal micronutrient deficiency induced changes in rat offspring [11], [14], [15], oxidative stress was determined in the liver by measuring lipid peroxidation (Malondialdehyde), protein carbonyls, reduced/oxidized glutathione (GSH/GSSG) and activity of catalase in post mitochondrial supernatant (20,000 g supernatant) as described earlier [36]. Activities of superoxide dismutase (SOD) and glutathione peroxidase were determined in the cytosol fraction according to Padmavathi et al [14].

### Statistical analysis

The results are presented as mean ± SEM and the data was analyzed using SPSS statistics package (version 21.0). Values for different parameters in the offspring are given only at 3 and 12 months of age for simplicity. All parameters in B12R female rats (before mating) and their pups were compared with respective controls. Differences between control and B12R female Wistar rats (just before mating) were analyzed using the Student’s ‘t’ test. One-way ANOVA followed by post hoc least significant difference test was used to analyze the differences among the offspring of different groups at a given time point. Differences were considered significant if ‘p’ was at least ≤0.05.

## Results

### Effects in female Wistar rats

Food intake was comparable among control and B12R diet fed female Wistar rats. (Table 1). After 3 months of feeding, B12R rats had ∼75% lower levels of plasma vitamin B12 and significantly higher plasma homocysteine levels than control rats. Although B12R rats weighed significantly heavier than controls at the end of 3 months of feeding, they had lower LBM% and FFM% than controls. However, TAF% (Table 1) and reproductive performance were comparable between the two groups of rats. (Table S1).

**Table 1:** Food intake, plasma vitamin B12, homocysteine concentrations and body composition in female rats after 3 months of Feeding.

Parameter	Control	B12R
**Food intake (g/day)**	12.9±0.466	13.5±0.340
**Vitamin B12 (pg/ml)**	1164±8.1	277±4.54*
**Homocysteine (µM)**	4.89±0.358	8.03±0.208*
**Body weight (g)**	184±4.46	213±7.82*
**LBM (%)**	90.5±0.919	87.5±0.685*
**FFM (%)**	57.3±0.764	53.6±0.752*
**TAF (%)**	33.2±0.277	33.7±0.321

Food intake, plasma vitamin B12 and homocysteine concentrations, body weights, percentages of (Lean Body Mass) LBM, (Fat Free Mass) FFM and Tissue Associated Fat (TAF) in Wistar female rats fed control and vitamin B12 restricted (B12R) diets for 3 months from weaning. Values are mean ± SE (n = 6).

*Significantly different from controls by Student’s ‘t’ test (p<0.05).

### Effects in offspring

#### Body weight at birth, weaning and later

At birth, B12R offspring weighed significantly lower than controls and rehabilitation from conception (B12RC) prevented this change (Table 2). Interestingly B12R offspring weighed significantly higher than controls at/from weaning, whereas the weaning weights of B12RC, B12RP offspring were comparable to that of control offspring (Table 2). Food intake was comparable among all the groups at all the time points studied (Table S2). B12RC and B12RP prevented the increase in offspring’s body weight (compared to B12R) at all the time points studied (Table 2), whereas rehabilitation from weaning (B12RW) mitigated the change *albeit* partly.

**Table 2:** Plasma vitamin B12, homocysteine concentrations and body weight changes in male offspring at various time points of age.

Group	Time point	Control	B12R	B12RC	B12RP	B12RW
**Vitamin B12 (pg/ml)**	3 months	1142±60.5^a^	32.2±11.6^b^	275±29.1^c^	301±18.5^c^	363±61.5^c^
	9 months	958±17.3^a^	132±11.3^b^	665±48.6^c^	589±29.7^c^	735±49.1^c^
	12 months	925±14.2^a^	87.0±7.44^b^	610±28.9^c^	673±11.3^c^	741±15.4^c^
**Homocysteine (µM)**	12 months	12.6±1.80	18.5±2.52	12.7±2.13	13.2±0.955	16.0±3.55
**Body weight (g)**	Birth	6.41±0.407^a^	5.61±0.212^b^	6.15±0.241^a^	-	-
	Weaning	36.0±2.72^a^	51.1±4.64^b^	35.4±2.51^a^	36.0±2.00^a^	51.1±4.64^b^
	3 months	227±13.7^a^	356±5.42^b^	165±9.36^c^	223±19.2^a^	283±6.66^d^
	9 months	390±16.1^a^	546±19.4^b^	398±13.7^a^	431±10.1^c^	446±4.51^c^
	12 months	391±18.8^a^	577±9.93^b^	418±15.5^a^	406±17.5^a^	431±10.4^a^

Plasma vitamin B12 and homocysteine concentrations and body weight changes in male offspring fed different diets at different time points; control, B12 restriction (B12R), B12 rehabilitation from conception (B12RC), B12 rehabilitation from parturition (B12RP), B12 rehabilitation from weaning (B12RW). Values are mean ± SE (n = 6). **Values in a row with different superscripts (a/b/c/d) are significantly different from others at p<0.05 by one way ANOVA/LSD tests.**

#### Plasma Vitamin B12 and homocysteine

As expected, B12R offspring had significantly lower (than control) levels of plasma vitamin B12 till their sacrifice at 12 months of age (Table 2). Although B12RC, B12RP and B12RW offspring had higher levels of plasma vitamin B12 than B12R, their levels were significantly lower than those of controls at all time-points studied (Table 2). Though higher in B12R offspring than controls, plasma homocysteine levels were comparable between the two groups at 12 months of age. As a corollary, plasma homocysteine levels in the rehabilitation groups were also comparable to those of control and B12R offspring at 12 months of age (Table 2).

#### Body composition

**Figure 2:**
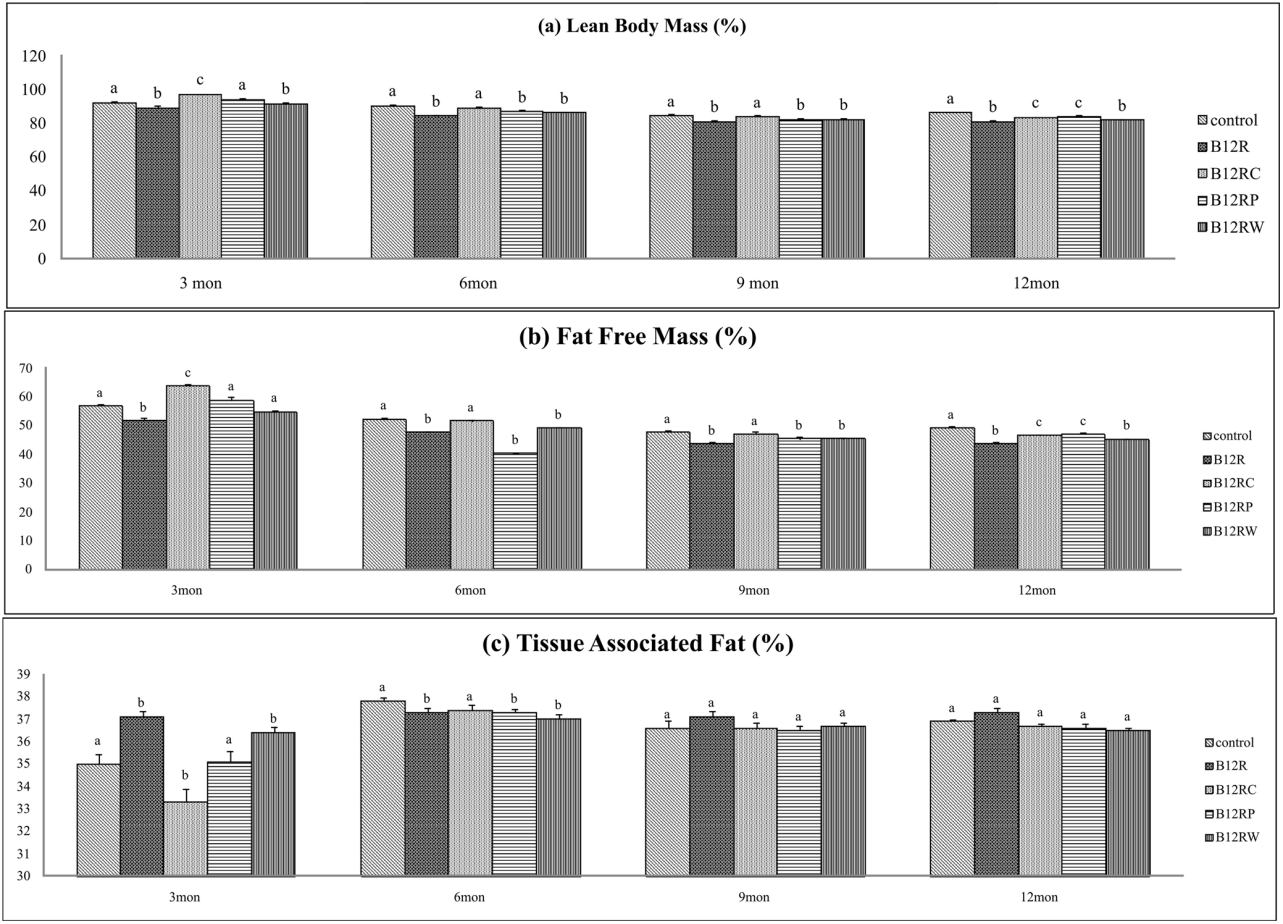
Body composition changes in male offspring. Body composition changes at different time points in Wistar rat male offspring fed different diets: Control (C), B12 restricted (B12R), B12 rehabilitation from conception (B12RC), B12 rehabilitation from parturition (B12RP) and B12 rehabilitation from weaning (B12RW). (a) Lean Body Mass%, (b) Fat Free Mass% (c) Tissue Associated fat%. Values are mean ± SE (n = 6). Bars with different superscripts (a/b/c) are significantly different from one another at p<0.05 by one way ANOVA/LSD test.

Both at three and twelve months of their age, LBM% and FFM% of B12R offspring were lower than those of controls (Figure 2). Although all three rehabilitation regimes appeared to correct these changes at three months of age, only B12RC and B12RP but not B12RW appeared to do so at twelve months of age. These findings suggest the importance of dams’ vitamin B12 status during pregnancy and lactation in programming/modulating the body composition of the offspring.

TAF% was higher in B12R than control offspring at 3 months of age; B12RC and B12RP but not B12RW appeared to correct this change at this time point. However, TAF% was comparable among groups at twelve months of age, probably suggesting the transient nature of the change.

#### Glucose uptake by diaphragm

For reasons mentioned earlier (materials and methods section) diaphragm was used in this study to determine the effects if any, of maternal vitamin B12 status on glucose uptake (basal and insulin stimulated) by skeletal muscle in the offspring. No significant difference was observed among different groups either in the basal or insulin stimulated glucose uptake by diaphragm (muscle) at any of the time points studied, suggesting that maternal vitamin B12 status may not affect the function of the muscle (glucose uptake). (Table S3).

#### Oral glucose tolerance and insulin response to glucose challenge

**Figure 3:**
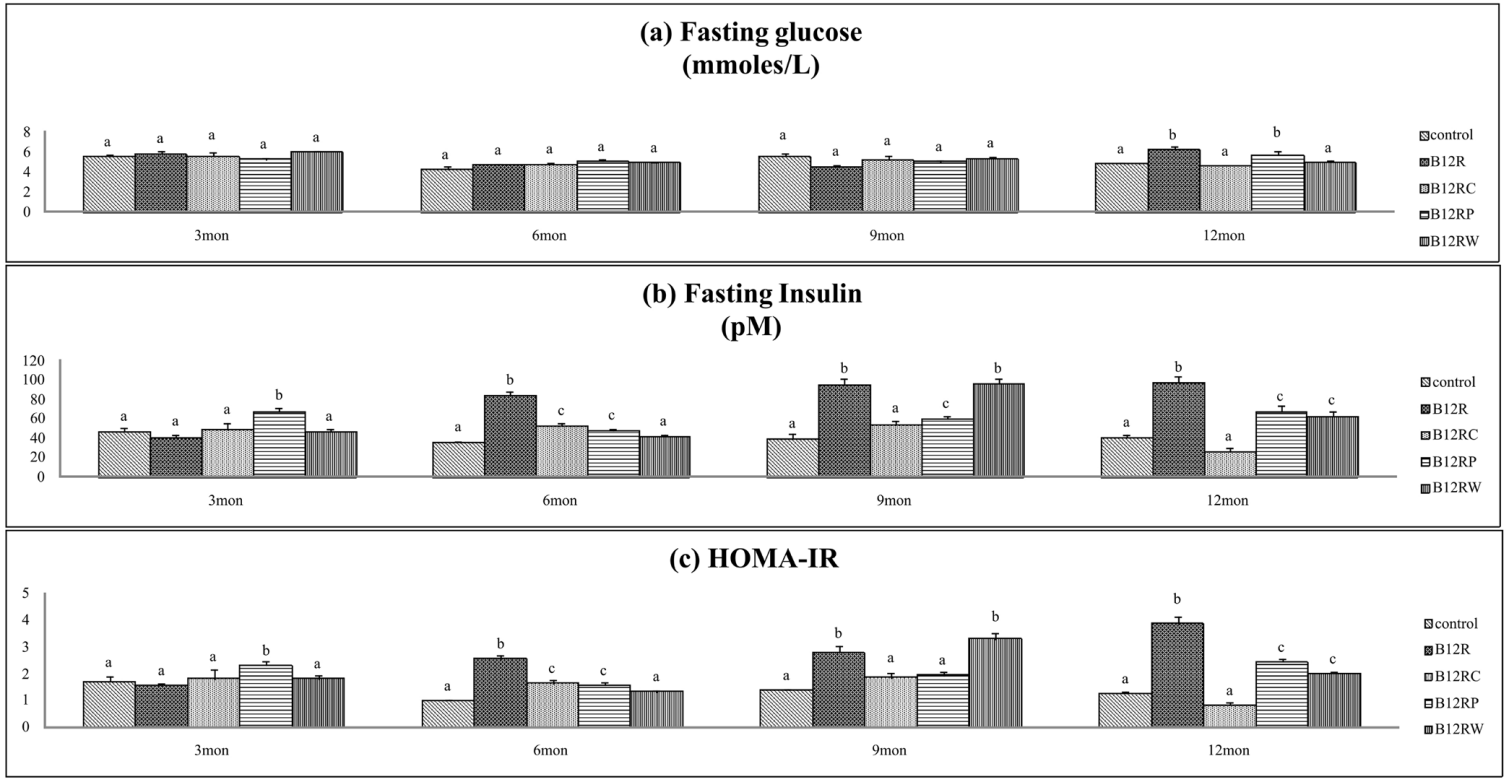
Glucose tolerance and insulin resistance parameters in male offspring. Glucose tolerance and insulin resistance parameters at different time points in Wistar rat male offspring fed different diets: Control (C), B12 restricted (B12R), B12 rehabilitation from conception (B12RC), B12 rehabilitation from parturition (B12RP) and B12 rehabilitation from weaning (B12RW). (a) Fasting glucose (mmol/L), (b) Fasting insulin (pM), (c) HOMA-IR. Values are mean ± SE (n = 6). Bars with different superscripts (a/b/c) are significantly different from one another at p<0.05 by one way ANOVA/LSD test.

Fasting hyperglycemia and hyperinsulinemia were seen in B12R offspring at 12 months of age (but not earlier). Vitamin B12 rehabilitation from conception mitigated these changes whereas rehabilitation from parturition and weaning did so *albeit* partly (Figure 3). Accordingly, HOMA IR was higher (than controls) in B12R offspring at twelve months of age and B12RC restored the values to control levels, whereas B12RP and B12RW could correct the change only partially (Figure 3).

**Figure 4:**
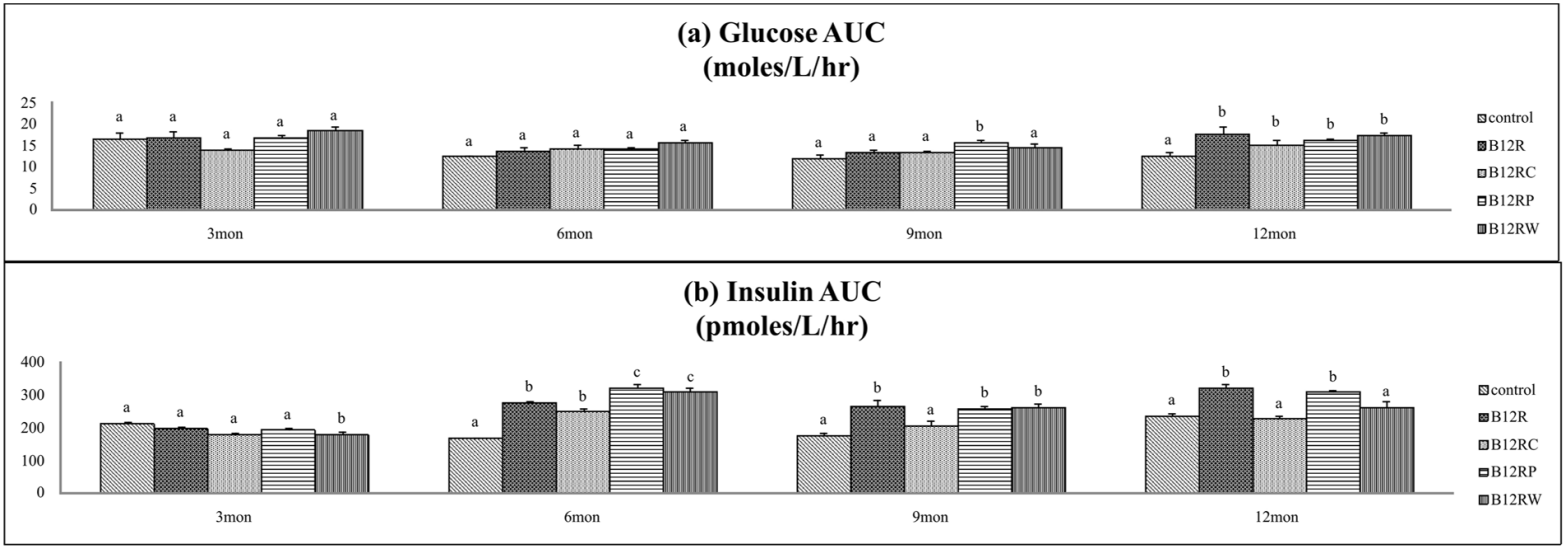
Glucose and insulin AUC during oral glucose tolerance test. Area Under Curves (AUC) of Glucose and insulin at different time points in Wistar rat male offspring fed different diets: Control (C), B12 restricted (B12R), B12 rehabilitation from conception (B12RC), B12 rehabilitation from parturition (B12RP) and B12 rehabilitation from weaning (B12RW). (a) Glucose AUC (moles/L/hr), (b) Insulin AUC (pmoles/L/hr). Values are mean ± SE (n = 6). Bars with different superscripts (a/b/c) are significantly different from one another at p<0.05 by one way ANOVA/LSD test.

In line with fasting hyperglycemia and hyperinsulinemia B12R offspring had higher (than controls) AUC for glucose and insulin during the oral glucose tolerance test, at 12 months of age but not earlier. While none of the rehabilitation regimes corrected the increase in AUC glucose during the OGTT (Figure 4), the increased insulin AUC was reversed by B12RC, but the mitigation was only partial by B12RP and B12RW (Figure 4). The ratio of AUC glucose and AUC insulin was comparable among groups at all-time points tested (Table S4).

#### Hepatic gluconeogenic enzyme activities

Phosphoenolpyruvate-carboxykinase (PEPCK) activity was higher (than controls) in B12R offspring; B12RC and B12RP but not B12RW corrected this change (Table 3). Increased activity of fructose-1-6-bisphosphotase observed in B12R offspring was corrected partially by B12RC and B12RW but it was curious that B12RP could reverse this change. Interestingly, the activities of Glucokinase and Glucose-6-phosphotase were comparable among all the groups. However, a decreased activity of pyruvate kinase was observed in B12R offspring and this was corrected by B12RC and B12RP but not by B12RW (Table 3).

**Table 3:** Specific activities of rate limiting enzymes involved in carbohydrate metabolism in male offspring.

Enzyme (units/ml/mg protein)	Control	B12R	B12RC	B12RP	B12RW
**Phosphoenol-pyruvate-carboxykinase**	1.54±0.253^a^	3.47±0.604^b^	1.68±0.221^a^	2.61±0.609^a^	3.07±0.309^b^
**Fructose-1,6-bisphosphotase**	1.26±0.114^a^	2.99±0.519^b^	2.28±0.131^c^	1.23±0.209^a^	2.00±0.138^c^
**Glucose-6-phosphotase**	3.17±0.250	3.22±0.129	3.21±0.132	3.86±0.439	3.39±0.118
**Glucokinase**	1.22±0.283	1.08±0.209	1.16±0.237	1.12±0.200	1.02±0.093
**Pyruvatekinase**	3.11±0.249^a^	1.54±0.278^b^	2.96±0.786^a^	2.61±0.512^a^	1.30±0.341^b^

Specific activities of rate limiting enzymes involved in carbohydrate metabolism in male offspring fed different diets at 12 months of age; control, B12 restriction (B12R), B12 rehabilitation from conception (B12RC), B12 rehabilitation from parturition (B12RP), B12 rehabilitation from weaning (B12RW). **Values are mean** ± **SE (n = 6). Values in a row with different superscripts (a/b/c) are significantly different from one another at p<0.05 by one way ANOVA/LSD tests.**

#### Oxidative Stress in B12R rats

Increased protein carbonyl and MDA levels were observed in the liver of B12R compared to controls. The changes were corrected *albeit* partially, by the rehabilitation regimes. Decreased concentration of reduced glutathione and increased concentration of oxidised glutathione were observed in B12R rat offspring (compared to controls) and these were also corrected partially by B12 rehabilitation. Interestingly, the ratio of reduced and oxidised glutathione (GSH/GSSG) that was significantly lowered in B12R was restored to control levels by B12RC alone, whereas the other rehabilitations showed only partial correction of the change (Table 4).

**Table 4:** Oxidative stress markers, specific activity of antioxidant enzymes in liver tissues of male offspring at 12 months of their age.

Parameter	Control	B12R	B12RC	B12RP	B12RW
**Proteincarbonyls (nmoles/mg protein)**	1.05±0.156^a^	2.77±0.148**^b^**	1.92±0.405**^c^**	1.83±0.366^a^	2.02±0.445**^b^**
**Malondialdehyde (nmoles/mg protein)**	0.413±0.079^a^	0.990±0.135**^b^**	0.847±0.222**^b^**	0.751±0.195^a^	0.524±0.108^a^
**Reduced glutathione (µmol/mg protein)**	0.764±0.097^a^	0.278±0.035**^b^**	0.605±0.082^a^	0.652±0.097^a^	0.521±0.100**^c^**
**Oxidised glutathione (µmol/mg protein)**	4.05±0.165^a^	6.81±0.311**^b^**	3.42±0.195^a^	5.68±0.249**^c^**	5.61±0.170**^c^**
**Reduced glutathione (GSH)/oxidized glutathione (GSSG)**	0.191±0.027^a^	0.041±0.005**^b^**	0.174±0.020^a^	0.112±0.012**^c^**	0.092±0.018**^c^**
**Superoxidedismutase (units/ml/mg protein)**	0.820±0.057^a^	0.302±0.045**^b^**	0.743±0.052^a^	0.707±0.101^a^	0.987±0.182^a^
**Glutathioneperoxidase (units/ml/mg protein)**	0.223±0.020	0.238±0.038	0.228±0.027	0.220±0.023	0.294±0.039
**Catalase (units/ml/mg protein)**	0.079±0.006^a^	0.030±0.003**^b^**	0.055±0.008^a^	0.062±0.018^a^	0.055±0.006^a^

Oxidative stress and specific activities of antioxidant enzymes in male offspring fed different diets at 12 months of age; control, B12 restriction (B12R), B12 rehabilitation from conception (B12RC), B12 rehabilitation from parturition (B12RP), B12 rehabilitation from weaning (B12RW). Values are mean ± SE (n = 6). **Values in a row with different superscripts (a/b/c) are significantly different from others at p<0.05 by one way ANOVA/LSD tests.**

A significant decrease was observed in the activity of superoxide dismutase (SOD) and catalase in B12R rats and this was corrected/restored to control levels by all three rehabilitation regimes. However, glutathione peroxidase activity was comparable among all the groups (Table 4).

## Discussion

Micronutrients are well known to be associated with metabolic diseases like Type 2 diabetes, Coronary artery disease etc. We had shown earlier that chronic dietary vitamin B12 restriction *per se* increased the body weight and body fat specially the visceral adiposity [8] in female Wistar rats. We now report here for the first time to the best of our knowledge that chronic dietary vitamin B12 restriction *per se* decreased the % of LBM and FFM without affecting their TAF%. Taken together with our earlier report the present findings indicate that chronic vitamin B12 restriction in rats increased only central adiposity but not tissue associated fat, implying that the decrease observed in LBM% and FFM% could be due to decreased soft tissue (muscle?) mass.

In line with decreased litter size and birth weight reported earlier in the offspring of vitamin B12 deficient mothers [3], maternal vitamin B12 restriction decreased the birth weight of the offspring highlighting the importance of maternal vitamin B12 status in fetal development. Our findings are in line with recent studies demonstrating strong association between vitamin B12 and intrauterine growth retardation [43] and the influence of erythrocyte folate and serum vitamin B12 status on birth weight [44]. They also agree with the Pune maternal nutritional study which demonstrated micronutrient deficiencies to be important limiting factors for fetal growth in undernourished communities [45]. That rehabilitation of pregnant B12R dams from conception restored the offspring’s birth weight to controls appears to validate this inference and is in agreement with the report that maternal iron supplementation improved the birth weight [46].

Rehabilitating vitamin B12 restricted dams from conception/parturition or their offspring from weaning improved the plasma vitamin B12 levels of the offspring, but the levels were lower than those of controls even at twelve months of their age, perhaps suggesting that vitamin B12 deficiency created in dams may not be mitigated completely even on long term rehabilitation. Nevertheless, rehabilitation could prevent the marginal increase in plasma homocysteine levels in B12R offspring, perhaps be due to the fact that there was no concurrent folate deficiency in the animals. Although the higher body weights (than controls) of B12R dams and their offspring from weaning onwards seems to suggest greater feed efficiency in B12R rats, our finding that the increased weight was associated with increased body fat rather than LBM or FFM negates such an inference.

Abundant literature indicates altered body adiposity and lipid metabolism to be the earliest changes seen, much before tissue insulin resistance manifests [47], [48]. We reported earlier that although the birth weight in B12R pups were lower than the controls, the body weight increased with age and was significantly higher than that of controls at 12 months. Further, the increased body weight of the B12R offspring was associated with increased body fat percentage (especially central adiposity) [8]. We now report the decrease in the % of LBM and FFM (but not TAF%) in the B12R dams and offspring suggesting that the decreased LBM% and FFM% could indeed be due to an absolute decrease in the soft tissue (muscle?) mass. Considering that adipose and muscle are the two insulin sensitive tissues primarily responsible for the postprandial clearance of glucose [23], it is evident from these results that maternal vitamin B12 restriction altered the offspring’s body composition (specially of adipose and muscle) in a way suggestive of their predisposal to insulin resistance in later life. It was interesting that though maternal vitamin B12 restriction decreased LBM% and FFM% suggestive of decreased muscle mass, it did not affect the muscle function as indicated by our finding that basal or insulin stimulated glucose uptake by the muscle (diaphragm) were not affected in B12R offspring.

Our findings are in line with the following literature: i) Skeletal muscle develops in the early and mid-gestation and nutritional insult at this stage might impair muscle development affecting muscle fiber number and composition, which may predispose the offspring to obesity and type 2 diabetes [49], ii) Nutrient deficiencies in ruminants during midgestation reduce the secondary muscle fiber formation [10] and iii) Children born to vitamin B12 deficient mothers were reported to have a reduced cardiac sympathetic activity [50]. Considering that up-regulation of Wnt/β-catenin pathway promotes myogenesis and down-regulation enhances adipogenesis [10], it will be interesting to decipher the role if any of this mechanism in mediating the maternal Vitamin B12 restriction induced changes in adipose and muscle development in the offspring. That changes in LBM% and FFM% were at least partially corrected by B12RC and B12RP but not by B12RW again seems to confirm the importance of vitamin B12 during pregnancy and lactation in determining/programming the soft tissue development in the offspring. This finding is in line with a previous report that maternal zinc supplementation improved lean tissue mass accretion in the offspring [51]. It is also supported by the report that maternal folate supplementation reduced the risk of developing cardiovascular diseases in their offspring [52].

Maternal vitamin B12 deficiency is associated with gestational diabetes and later T2D in mothers and offspring [53]. Yajnik et al [54] proposed that low maternal vitamin B12 with high folate levels could be an important contributor to the epidemic of adiposity and type 2 diabetes in India. In line with these reports maternal vitamin B12 restriction induced fasting hyper-insulinemia and insulin resistance (increased HOMA-IR) in the offspring as early as six months of age, whereas fasting hyper-glycemia and impaired glucose tolerance were observed at 12 months of age. Indeed at 12 months of age, B12R offspring were insulin resistant both under fasting (high HOMA IR) and fed conditions (higher AUC insulin during OGTT). These observations are also in agreement with reports that maternal vitamin D deficiency was associated with poor muscle mass and higher insulin resistance in their offspring [55]. The importance of maternal vitamin B12 status in modulating insulin sensitivity/resistance in the offspring and their probable causal relationship is apparent from the finding that the changes were corrected by B12RC. Further, these findings corroborate our recent observations in maternal Cr restricted offspring [14] and partly agree partially with those in MgR offspring [12].

That the fasting hyperglycemia in B12R offspring could be due to altered intracellular glucose metabolism was studied next. Indeed, the increase in the activities of phosphoenolpyruvate-carboxykinase (PEPCK) and fructose-1-6-bisphosphotase in the liver of the B12R rat offspring appears to suggest increased gluconeogenesis in B12R offspring. That activity of PEPCK was reversed by B12RC but only partly by B12RP and B12RW, whereas that of fructose-1-6-bisphosphotase was reversed by B12RP and partly by B12RC and B12RW, not only suggest their causal relationship but also the differential effects of vitamin B12 on these enzymes of gluconeogenic pathway. In line with these findings, there was a decreased activity of *pyruvatekinase* in B12R offspring and was reversed by B12RC and B12RP but not by B12RW. It was however intriguing that glucokinase and glucose-6-phosphatase activities were unaffected in B12R offspring. These findings thus reiterate the importance of maternal vitamin B12 status during pregnancy and lactation in modulating/programming intracellular glucose metabolism (e.g., increasing gluconeogenesis) in the offspring.

Inflammation and oxidative stress play a key role in the development of adiposity in the offspring, while the restoration of antioxidant status improved the condition [56]. Oxidative stress is proposed to be a mechanism through which obesity, insulin resistance and metabolic syndrome lead to T2DM in humans [42]. Considering that maternal dietary restriction of a variety of micronutrients resulted in similar phenotypic changes in rat offspring [57], the present findings of similar nature in B12R offspring prompted us to decipher the probable associated/underlying common mechanism(s). The observed increase in the levels of MDA, protein carbonyls, oxidized glutathione and decreased levels of reduced glutathione along with decreased activities of SOD and catalase strongly indicate that increased oxidative stress probably due to decreased antioxidant capacity could be a common underlying/associated mechanism. That these changes also were corrected by rehabilitation *albeit* partially probably suggest a causal relationship between maternal vitamin B12 status and oxidative stress in the offspring. Unlike the SOD and catalase activities, glutathione-peroxidase activity was increased in B12R offspring corroborating the differential modulation of antioxidant enzyme activities by maternal vitamin B12 restriction, probably to cope up with increased oxidative stress. These findings are in general agreement with earlier observations in the offspring of various vitamin and mineral restricted rats [11], [14], [15], [58]–[62].

We recently identified 38 differentially expressed proteins in the liver of B12R offspring that were enriched in pathways regulating amino acid, lipid and carbohydrate metabolism [63] and also reported the age dependent differential expression of peroxisome proliferator activated receptor (PPAR) α and γ in the liver of B12R pups. Vitamin B12, folic acid and DHA play an important role in one carbon metabolism and influence global DNA methylation in placenta [64] altering gene expression. Thus it seems possible that the changes observed in the expression of various proteins/genes could be due to the modulation of epigenetics by the maternal vitamin B12 status. Indeed Ian et al [65] demonstrated the reversal of maternally inherent epigenomic marks through methyl supplementation in adult offspring suggesting the importance of one carbon metabolic pathway and hence vitamin B12 status. Studies are in progress in B12R offspring to assess the epigenetic changes if any that underlie the altered gene expression and their modulation by rehabilitation.

In conclusion, maternal vitamin B12 restriction decreased the % of LBM and FFM in the offspring but had no effect on their TAF% or glucose uptake (basal and insulin stimulated) by the muscle. Nevertheless the offspring were insulin resistant, impaired glucose tolerant and increased hepatic gluconeogenesis appeared to be a mechanism underlying fasting hyperglycemia. Increased oxidative stress perhaps due to reduced anti-oxidant enzyme activity seems to underlie the effects of maternal vitamin B12 restriction, while differential regulation of gene expression by modulating epigenetics may be another mechanism. That rehabilitation from conception and parturition but not weaning, corrected maternal vitamin B12 restriction induced changes (including those in oxidative stress) in the offspring not only highlight the role of oxidative stress in mediating these changes but also the importance of vitamin B12 in fetal programming of the body composition and glucose metabolism in their later life. Our findings are in line with the hypothesis that improved environmental conditions (e.g., maternal nutrition) would eventually lead to a decline in the prevalence of type 2 diabetes and other cardiovascular risk factors, as better maternal health and nutrition result in improved fetal growth [66].

## Supporting Information

Table S1 **Reproductive Performance of Wistar female rats fed control and vitamin B12 restricted diets (B12R) for 3 months from weaning.**
(DOCX)

Table S2 **Food intake (grams/day) by male offspring at different time points.** Food intake by male offspring at different time points; control, B12 restriction (B12R), B12 rehabilitation from conception (B12RC), B12 rehabilitation from parturition (B12RP), B12 rehabilitation from weaning (B12RW). Values are mean ± SE (n = 6).
(DOCX)

Table S3 **Glucose uptake by diaphragm (nmol/hour/gram tissue) of male offspring of different groups at 12 months of age.** Glucose uptake by diaphragm in male offspring at 12 months of age. Control, B12 restriction (B12R), B12 rehabilitation from conception (B12RC), B12 rehabilitation from parturition (B12RP), B12 rehabilitation from weaning (B12RW). Values are mean ± SE (n = 6).
(DOCX)

Table S4 **AUC glucose: AUC insulin ratio in the offspring during oral glucose tolerance test.** Area Under Curve (AUC) ratio of glucose and insulin at different time points of their age. Control, B12 restriction (B12R), B12 rehabilitation from conception (B12RC), B12 rehabilitation from parturition (B12RP), B12 rehabilitation from weaning (B12RW). Values are mean ± SE (n = 6). Values in a column (a,b) with different superscripts are significantly different from one another at p<0.05 by one way ANOVA/LSD tests.
(DOCX)
