# Supplementary material for: Chronic Maternal Vitamin B12 Restriction Induced Changes in Body Composition & Glucose Metabolism in the Wistar Rat Offspring Are Partly Correctable by Rehabilitation
Source: PLoS One. 2014 Nov 14;9(11):e112991. doi: 10.1371/journal.pone.0112991 (PMC4232526; doi:10.1371/journal.pone.0112991)
Supplement: Table S1 — Reproductive Performance of Wistar female rats fed control and vitamin B12 restricted diets (B12R) for 3 months from weaning. (DOCX) [file pone.0112991.s001.docx]

**Supporting Table S1:**

**Reproductive Performance of Wistar female rats fed control and vitamin B12 restricted diets (B12R) for 3 months from weaning**

| **Nature of diet** | **Control** | **B12R** |
| --- | --- | --- |
| **Animals/group** | **6** | **24** |
| **Body wt. Before mating (g)** | **210** | **225** |
| **%Conceived** | **100** | **95.8** |
| **Wt. Gain during pregnancy (g)** | **44.4** | **49.7** |
| **%Aborted** | **0** | **4.16** |
| **Litter size** | **11** | **12** |
| **Total pups** | **66** | **288** |
| **Still births** | **0** | **7** |
| **% Still birth** | **0** | **2.43** |
| **No. of males** | **31** | **154** |
| **No. of females** | **35** | **134** |
| **Litter size during Lactation/group** | **8** | **8** |
| **Deaths during lactation** | **2** | **16** |
| **% Deaths during lactation** | **3.0** | **5.6** |
