# Supplementary material for: Chronic Maternal Vitamin B12 Restriction Induced Changes in Body Composition & Glucose Metabolism in the Wistar Rat Offspring Are Partly Correctable by Rehabilitation
Source: PLoS One. 2014 Nov 14;9(11):e112991. doi: 10.1371/journal.pone.0112991 (PMC4232526; doi:10.1371/journal.pone.0112991)
Supplement: Table S2 — Food intake (grams/day) by male offspring at different time points. Food intake by male offspring at different time points; control, B12 restriction (B12R), B12 rehabilitation from conception (B12RC), B12 rehabilitation from parturition (B12RP), B12 rehabilitation from weaning (B12RW). Values are mean ± SE (n = 6). (DOCX) [file pone.0112991.s002.docx]

**Supporting Table S2:**

**Food intake (grams/ day) by male offspring atdifferent time points**

| **Group** | **Food intake**  **(3 months)** | **Food intake**  **(6 months)** | **Food intake**  **(9 months)** | **Food intake**  **(12 months)** |
| --- | --- | --- | --- | --- |
| **Control** | 14.1 ± 0.372 | 16.3 ± 0.332 | 17.9 ± 0.368 | 20.8 ± 0.524 |
| **B12R** | 13.9 ± 0.332 | 16.1 ± 0.257 | 17.3 ± 0.237 | 20.7 ± 0.400 |
| **B12RC** | 14.1 ± 0.192 | 16.3 ± 0.288 | 17.8 ± 0.347 | 21.2 ± 0.359 |
| **B12RP** | 13.7 ± 0.351 | 16.6 ± 0.227 | 18.1 ± 0.381 | 21.0 ± 0.371 |
| **B12RW** | 13.6 ± 0.202 | 16.4 ± 0.194 | 17.7 ± 0.315 | 20.7 ± 0.370 |

Food intake by male offspring at different time points; Control, B12 restriction (B12R), B12 rehabilitation from conception (B12RC), B12 rehabilitation from parturition (B12RP), B12 rehabilitation from weaning (B12RW). Values are mean SE (n=6).
