# Supplementary material for: Chronic Maternal Vitamin B12 Restriction Induced Changes in Body Composition & Glucose Metabolism in the Wistar Rat Offspring Are Partly Correctable by Rehabilitation
Source: PLoS One. 2014 Nov 14;9(11):e112991. doi: 10.1371/journal.pone.0112991 (PMC4232526; doi:10.1371/journal.pone.0112991)
Supplement: Table S3 — Glucose uptake by diaphragm (nmol/hour/gram tissue) of male offspring of different groups at 12 months of age. Glucose uptake by diaphragm in male offspring at 12 months of age. Control, B12 restriction (B12R), B12 rehabilitation from conception (B12RC), B12 rehabilitation from parturition (B12RP), B12 rehabilitation from weaning (B12RW). Values are mean ± SE (n = 6). (DOCX) [file pone.0112991.s003.docx]

**Supporting Table S3:**

**Glucose uptake by diaphragm (nmol/hour/gram tissue) of male offspring of different groups at 12 months of age**

| **Group** | | **12 months** |
| --- | --- | --- |
| **Control** | Insulin | 0.482 ± 0.032 |
|  | Basal | 0.405 ± 0.144 |
|  | Insulin/Basal | 1.46 ± 0.424 |
| **B12R** | Insulin | 0.436 ± 0.043 |
|  | Basal | 0.411 ± 0.073 |
|  | Insulin/Basal | 1.11 ± 0.142 |
| **B12RC** | Insulin | 0.406 ± 0.038 |
|  | Basal | 0.433 ± 0.064 |
|  | Insulin/Basal | 0.975 ± 0.114 |
| **B12RP** | Insulin | 0.407 ± 0.053 |
|  | Basal | 0.382 ± 0.034 |
|  | Insulin/Basal | 1.05 ± 0.077 |
| **B12RW** | Insulin | 0.526 ± 0.084 |
|  | Basal | 0.395 ± 0.015 |
|  | Insulin/Basal | 1.33 ± 0.233 |

Glucose uptake by diaphragm in male offspring at 12 months of age. Control, B12 restriction (B12R), B12 rehabilitation from conception (B12RC), B12 rehabilitation from parturition (B12RP), B12 rehabilitation from weaning (B12RW). Values are mean SE (n=6).
