# Supplementary material for: Chronic Maternal Vitamin B12 Restriction Induced Changes in Body Composition & Glucose Metabolism in the Wistar Rat Offspring Are Partly Correctable by Rehabilitation
Source: PLoS One. 2014 Nov 14;9(11):e112991. doi: 10.1371/journal.pone.0112991 (PMC4232526; doi:10.1371/journal.pone.0112991)
Supplement: Table S4 — AUC glucose: AUC insulin ratio in the offspring during oral glucose tolerance test. Area Under Curve (AUC) ratio of glucose and insulin at different time points of their age. Control, B12 restriction (B12R), B12 rehabilitation from conception (B12RC), B12 rehabilitation from parturition (B12RP), B12 rehabilitation from weaning (B12RW). Values are mean ± SE (n = 6). Values in a column (a,b) with different superscripts are significantly different from one another at p<0.05 by one way ANOVA/LSD tests. (DOCX) [file pone.0112991.s004.docx]

**Supporting Table S4:**

**AUC glucose : AUC insulin ratio in the offspring during oral glucose tolerance test**

| Group | 3 Months | 6 Months | 9 Months | 12 Months |
| --- | --- | --- | --- | --- |
| Control | 0.077 ± 0.009 | 0.074 ± 0.006 ^a^ | 0.070 ± 0.004 ^a^ | 0.050 ±0.006 |
| B12R | 0.085 ± 0.006 | 0.049 ± 0.003 ^b^ | 0.050 ± 0.002 ^b^ | 0.060 ± 0.004 |
| B12 RC | 0.078 ± 0.002 | 0.058 ± 0.005 ^a^ | 0.070 ± 0.004 ^a^ | 0.067 ± 0.005 |
| B12 RP | 0.087 ± 0.005 | 0.043 ± 0.001 ^b^ | 0.060 ± 0.003 ^b^ | 0.053 ± 0.002 |
| B12 RW | 0.104 ± 0.007 | 0.052 ± 0.003 ^b^ | 0.060 ± 0.006 ^b^ | 0.068 ± 0.006 |

Area Under Curve (AUC) ratio of glucose and insulin at different time points of their age. Control, B12 restriction (B12R), B12 rehabilitation from conception (B12RC), B12 rehabilitation from parturition (B12RP), B12 rehabilitation from weaning (B12RW). Values are mean SE (n = 6). Values in a column with different superscripts (a,b) are significantly different from one another at p < 0.05 by one way ANOVA / LSD tests.
